# Assessment of pneumoconiosis by dusty job type among underground coal miners participating in the Coal Workers’ Health Surveillance Program,1991–2024

**DOI:** 10.3389/fpubh.2026.1970741

**Published:** 2026-10-20

**Authors:** Nirmala T. Myers, David J. Blackley, Laura Reynolds, A. Scott Laney, Noemi B. Hall

**Affiliations:** Surveillance Branch, Respiratory Health Division, National Institute for Occupational Safety and Health, Centers for Disease Control and Prevention, Morgantown, WV, United States

**Keywords:** black lung, CMO, CWHSP, pneumoconiosis, roof bolter, tenure

## Abstract

**Introduction:**

Exposure to respirable coal mine dust causes coal workers’ pneumoconiosis (CWP). Despite regulatory efforts, progressive massive fibrosis (PMF), the most severe form of pneumoconiosis persists, particularly in Central Appalachia. This study evaluated pneumoconiosis prevalence among underground coal miners by occupation during 1991–2024.

**Methods:**

The study analyzed data from 30,319 underground miners in the Coal Workers Health Surveillance Program (CWHSP) from 1991–2024. Pneumoconiosis was identified from chest radiographs using standardized classifications. Disease prevalence was compared across mining occupations and geographic regions, with adjustment for underground mining tenure.

**Results:**

Continuous miner operators (CMOs) and roof bolters experienced the highest prevalence of disease. Compared with miners who never held these occupations, adjusted prevalence of CWP was higher among CMOs (PR = 1.8; 95% CI: 1.6–2.0) and roof bolters (PR = 1.4; 95% CI: 1.3–1.6). PMF prevalence was also elevated among CMOs (PR = 3.0; 95% CI: 2.3–4.0) and roof bolters (PR = 1.4; 95% CI: 1.1–1.9). CWP prevalence was 8.4% in Central Appalachia versus 1.5% in the rest of U. S., while PMF prevalence was 1.3% versus 0.1%. After adjustment for tenure, Central Appalachian miners had 3.9 times the prevalence of CWP (95% CI: 3.4–4.5) and 10 times the prevalence of PMF (95% CI: 5.7–17.5).

**Discussion:**

CMOs and roof bolters experienced the highest prevalence of pneumoconiosis, consistent with their classification as MSHA-designated “dusty” occupations. Markedly higher disease prevalence across all occupations in Central Appalachia indicates persistent hazardous dust exposures and underscores the need for continued prevention and surveillance efforts.

## Introduction

Coal mining has long been recognized as a hazardous occupation, with respirable coal mine dust exposure representing one of the most persistent and consequential occupational hazards in underground mining environments. Respirable coal mine dust is a heterogeneous mixture of coal, rock, and mineral particles, including respirable crystalline silica, and its composition varies according to mine geology, production methods, work location, and mining activities such as cutting, drilling, roof bolting, and haulage (1). Occupational exposure to respirable coal mine dust can result in a spectrum of severe respiratory diseases collectively referred to as “black lung.” This term encompasses coal mine dust-induced lung diseases, including coal workers’ pneumoconiosis (CWP), silicosis, mixed dust pneumoconiosis, dust-related diffuse fibrosis, and chronic obstructive pulmonary disease (COPD). Progressive massive fibrosis (PMF) is the most severe form of pneumoconiosis (2, 3). Respirable crystalline silica exposure has also been associated with increased risk for lung cancer and accelerated pneumoconiosis progression (4, 5).

Recognition of these health risks led to passage of the Federal Coal Mine Health and Safety Act of 1969 (Coal Act) and subsequent amendments establishing dust exposure limits and medical surveillance for coal miners (6, 7). Over time, the Mine Safety and Health Administration (MSHA) identified specific underground mining jobs associated with particularly high dust exposures. These jobs became known as “designated occupations.” Under the 2014 MSHA respirable dust rule (8), the category was expanded to include additional “other designated occupations.” These occupations include continuous miner operators (CMOs), longwall workers, roof bolters, shuttle car operators, and other occupations involved in coal extraction and transportation. Because these occupations are recognized as having elevated exposure potential, they are subject to enhanced respirable dust compliance sampling requirements.

Despite decades of regulatory efforts, severe pneumoconiosis persists among U. S. coal miners and has re-emerged in recent years, particularly in Central Appalachia (i.e., Kentucky, Virginia, and West Virginia) (9). Studies using MSHA compliance sampling data have demonstrated substantial differences in respirable dust exposure across underground mining occupations, with CMOs and roof bolters experiencing some of the highest measured coal dust exposures, including exposure to respirable crystalline silica generated during cutting and drilling through rock strata (10). Recent studies have documented increasing prevalence of advanced pneumoconiosis and PMF among working coal miners, particularly in Central Appalachia, underscoring the continuing burden of occupational lung disease despite existing dust control regulations (11–13).

Although occupational differences in respirable dust exposure are well documented, less is known about pneumoconiosis prevalence across specific underground mining occupations. The Coal Workers’ Health Surveillance Program (CWHSP) was established following the enactment of the 1969 Coal Act (6). For decades, the program has collected demographic, radiographic, and occupational history information from participating coal miners. This long-term data provides a unique opportunity to evaluate disease prevalence across mining occupations (14).

We examined the prevalence of CWP and PMF among underground coal miners participating in the CWHSP between 1991–2024, with a focus on occupations historically recognized as having elevated respirable dust exposure. Our objective was to characterize the differences in pneumoconiosis prevalence by occupation and geographic region among contemporary U. S. coal miners.

## Methods

Working coal miners who participated in the CWHSP from January 1, 1991, through December 31, 2024, were included in this analysis. Comprehensive job history details were not systematically collected prior to 1991. Because miners could have multiple CWHSP encounters during the study period, only the most recent eligible encounter for each miner was retained to capture the most current information on mining tenure, age, sex, geographic region of employment, occupational history, and radiographic classification. Unreadable radiographs and records from retired miners were excluded, leaving 37,274 unique miners. Of these, 6,955 miners with surface mining experience were excluded, resulting in a sample of 30,319 miners with only underground mining tenure. Data were missing only for sex (*n* = 8 miners) and ethnicity (*n* = 11,552 miners). Surface coal miners were evaluated previously (15). The Central Appalachian region was defined as the U. S. states of Kentucky, Virginia, and West Virginia (15).

Occupational history was captured utilizing the standardized, federally approved Miner Identification Document (Form CDC/NIOSH (M)2.9) (16). This questionnaire is administered at the initial CWHSP examination and collected at each subsequent periodic screening encounter (typically occurring every 3–5 years) (2). The form collects self-reported, free-text occupational history, including total years of underground coal mining, and most common job worked at each mine throughout their tenure (17). Occupations of interest (“dusty jobs”) from our data included those previously identified as having high exposure to respirable coal mine dust including respirable crystalline silica and are listed in Supplementary Table S1 (10). These occupation categories were not mutually exclusive because miners could report employment in multiple occupations during their mining careers. Miners were then categorically classified as ‘ever’ *vs*. ‘never’ employed in each respective job based on their lifetime work history reported up to that final encounter.

The presence of small opacities (profusion ≥1/0, according to the International Labor Office [ILO] Classification of Radiographs of Pneumoconiosis) (18) and/or large opacities (progressive massive fibrosis [PMF], defined as opacities >1 cm) was determined from chest radiographs, as described previously (19). Differences in age and miner tenure between groups were evaluated using the Wilcoxon rank-sum test. Associations between radiographic and mining tenure categories were evaluated using Pearson chi-square tests. Prevalence ratios (PRs) and 95% confidence intervals (CIs) were estimated using Poisson regression models with a log link, with underground mining tenure modeled continuously in years. CWP and PMF were evaluated as separate binary outcomes. For occupation-specific models, miners who had never worked in the occupation of interest served as the reference group. Geographic differences were evaluated by comparing miners in Central Appalachia with miners in other U. S. regions, with the latter serving as the reference group. Models were adjusted for underground mining tenure. Data were analyzed using SAS (version 9.4; SAS Institute, Cary, NC).

## Results

### Demographics

A total of 30,319 underground coal miners who participated in the CWHSP during 1991–2024 were included in this analysis. Participants were predominantly male (98.9%) and White (94.6%), with smaller proportions identifying as Black (2.9%), American Indian or Alaska Native (1.5%), Asian (0.9%), or other races (<0.1%). Among miners who reported ethnicity, 10.0% identified as Hispanic. Nearly half of participants worked in Central Appalachia (47.1%), followed by the rest of U. S in Eastern (21.6%), Interior (16.2%), and Western (15.1%) regions.

At most recent CWHSP encounter, the median age of miners was 44 years (range: 17–80; mean ± SD: 43 ± 11 years). Miners with CWP were older than those without disease (median: 51 *vs.* 43 years; mean ± SD: 51 ± 8 *vs.* 43 ± 11 years; *p* < 0.001). Median underground mining tenure was 17 years (range: 1–54; mean ± SD: 17 ± 11 years) overall and was longer in Central Appalachia than in other regions (median: 20 *vs*. 13 years; mean ± SD: 20 ± 11 *vs.* 15 ± 11 years; *p* < 0.001). Miners with CWP had substantially longer tenure than those without disease (median: 28 *vs.* 16 years; mean ± SD: 27 ± 9 *vs.* 17 ± 11 years; *p* < 0.001). Similarly, miners with PMF had a median age of 53 years (mean ± SD: 53 ± 7 years) and a median tenure of 32 years (mean ± SD: 31 ± 8 years).

### Pneumoconiosis

Increasing underground mining tenure was significantly associated with greater radiographic severity of pneumoconiosis (*p* < 0.01; Table 1). Most miners with CWP had category 1 ILO small opacity profusion (1/0–1/2); however, higher profusion categories (≥2/1) were more common among miners with longer tenure, particularly those with more than 25 years of underground mining.

**Table 1:** International labour organization (ILO) profusion category and progressive massive fibrosis (PMF) classification by underground mining tenure, CWHSP, 1991–2024^a^.

Underground mining tenure	1/0	1/1	1/2	2/1	2/2	2/3	3/2	3/3	3+	CWP Total	A	B	C	PMF Total
<10 years	17 (34.0%)	21 (42.0%)	5 (10.0%)	0 (0.0%)	2 (4.0%)	1 (2.0%)	4 (8.0%)	0 (0.0%)	0 (0.0%)	50	0 (0.0%)	1 (100.0%)	0 (0.0%)	1
10–14 years	19 (27.1%)	25 (35.7%)	16 (22.9%)	1 (1.4%)	6 (8.6%)	2 (2.9%)	1 (1.4%)	0 (0.0%)	0 (0.0%)	70	0 (0.0%)	3 (100.0%)	0 (0.0%)	3
15–19 years	23 (16.1%)	57 (39.9%)	34 (23.8%)	8 (5.6%)	14 (9.8%)	5 (3.5%)	1 (0.7%)	1 (0.7%)	0 (0.0%)	143	4 (40.0%)	5 (50.0%)	1 (10.0%)	10
20–24 years	53 (20.2%)	102 (38.8%)	49 (18.6%)	13 (4.9%)	27 (10.3%)	9 (3.4%)	7 (2.7%)	3 (1.1%)	0 (0.0%)	263	8 (25.0%)	16 (50.0%)	8 (25.0%)	32
≥ 25 years	159 (17.3%)	296 (32.1%)	183 (19.9%)	69 (7.5%)	88 (9.6%)	73 (7.9%)	21 (2.3%)	31 (3.4%)	1 (0.1%)	921	60 (40.3%)	67 (45.0%)	22 (14.8%)	149
Total	271 (18.7%)	501 (34.6%)	287 (19.8%)	91 (6.3%)	137 (9.5%)	90 (6.2%)	34 (2.3%)	35 (2.4%)	1 (0.1%)	1,447	72 (37.0%)	92 (47.2%)	31 (15.9%)	195

^a^Percentages for ILO profusion categories and PMF categories were calculated using row totals. Percentages may not sum to 100% because of rounding. The total miners with PMF (*n* = 195) were a subset of the 1,447 miners with CWP. PMF size categories were defined according to the ILO classification as A, one or more large opacities with a combined greatest dimension exceeding 10 mm but not exceeding 50 mm; B, one or more large opacities with a combined area greater than category A but not exceeding the area of the right upper lung zone; and C, one or more large opacities with a combined area exceeding the equivalent area of the right upper lung zone.

### CWP and PMF by mining occupation

The distribution of CWP also varied by mining occupation ever worked (Figure 1). Overall, 770 roof bolters and 663 CMOs had CWP, followed by 452 shuttle car operators and 367 laborers. Fewer than 100 cases of CWP were observed among other occupations including mechanics, motormen, drillers, and cutting machine operators. Similarly, 117 CMOs and 103 roof bolters had PMF.

**Figure 1:**
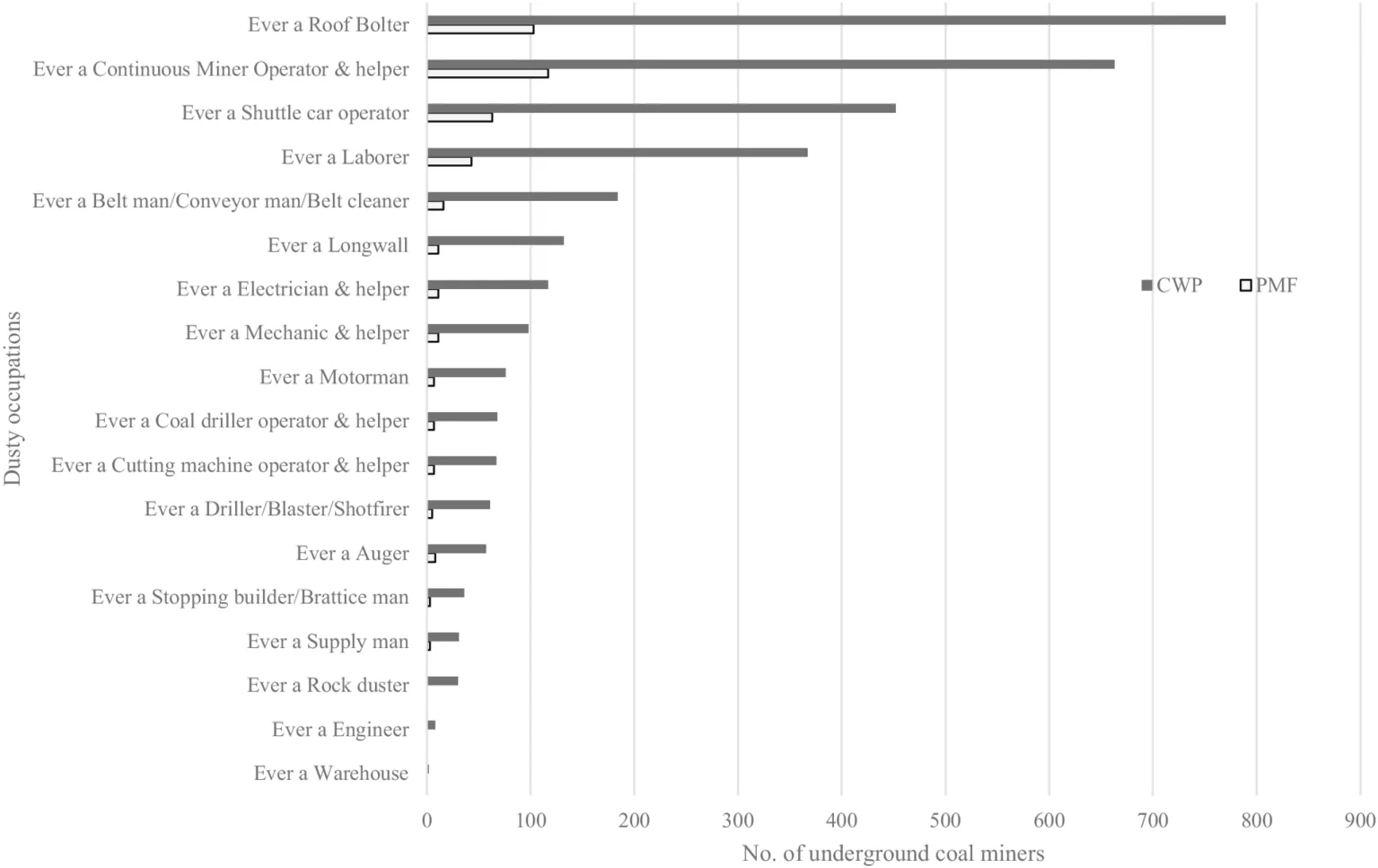
Coal Workers’ Pneumoconiosis (CWP) and Progressive Massive Fibrosis (PMF) by Mining Occupation in the Coal Workers’ Health Surveillance Program (CWHSP), 1991–2024. ^a^Miners were categorically classified as ‘ever’ vs. ‘never’ employed in each respective occupation based on their lifetime work history reported up to their final encounter with CWHSP.

### Prevalence in central Appalachia

Overall, the prevalence of both CWP and PMF was substantially higher in Central Appalachia compared with other U. S. regions. CWP prevalence was 8.4% in Central Appalachia versus 1.5% elsewhere (Table 2), while PMF prevalence was 1.3% versus 0.1%, respectively (Table 3). After adjustment for underground mining tenure, miners in Central Appalachia had nearly four times the prevalence of CWP (PR = 3.9; 95% CI: 3.4–4.5) and 10 times the prevalence of PMF (PR = 10.0; 95% CI: 5.7–17.5).

**Table 2:** Prevalence of coal workers’ pneumoconiosis (CWP) and associated prevalence ratios by region and occupational history among U. S. underground coal miners, CWHSP, 1991–2024.

	Coal workers’ pneumoconiosis (CWP)					
	*N*	*n* (%)	Unadjusted PR (95% CI)	*p*-value	Adjusted PR (95% CI)^a^	*p*-value
US underground coal miners						
Central Appalachia^b^	14284	1204 (8.4)	5.6 (4.9–6.4)	<0.001	3.9 (3.4–4.5)	<0.001
US excluding Central Appalachia	16035	243 (1.5)				
Roof bolter status						
Ever a roof bolter	13072	770 (5.9)	1.5 (1.4–1.7)	<0.001	1.4 (1.3–1.6)	<0.001
Never a roof bolter	17247	677 (3.9)				
Ever a roof bolter in Central Appalachia	7053	672 (9.5)	5.8 (4.7–7.2)	<0.001	4.4 (3.5–5.4)	<0.001
Ever a roof bolter in US excluding Central Appalachia	6019	98 (1.6)				
Continuous miner operator (CMO) status						
Ever a CMO	7871	663 (8.4)	2.4 (2.2–2.7)	<0.001	1.8 (1.6–2.0)	<0.001
Never a CMO	22448	784 (3.5)				
Ever a CMO in Central Appalachia	4011	574 (14.3)	6.2 (5.0–7.7)	<0.001	5.1 (4.1–6.3)	<0.001
Ever a CMO in US excluding Central Appalachia	3860	89 (2.3)				
Shuttle car operator status						
Ever a shuttle car operator	8641	452 (5.2)	1.1 (1.0–1.3)	0.018	0.9 (0.8–1.0)	0.040
Never a shuttle car operator	21678	995 (4.6)				
Ever a shuttle car operator in Central Appalachia	4819	399 (8.3)	6.0 (4.5–7.9)	<0.001	4.6 (3.5–6.2)	<0.001
Ever a shuttle car operator in US excluding Central Appalachia	3822	53 (1.4)				
Laborer status						
Ever a laborer	9430	367 (3.9)	0.8 (0.7–0.8)	<0.001	0.7 (0.6–0.8)	<0.001
Never a laborer	20889	1080 (5.2)				
Ever a laborer in Central Appalachia	4023	281 (7.0)	4.4 (3.5–5.6)	<0.001	3.3 (2.6–4.2)	<0.001
Ever a laborer in US excluding Central Appalachia	5407	86 (1.6)				
Belt man/Conveyor man/Belt cleaner (BMC) status						
Ever a BMC	4494	184 (4.1)	0.8 (0.7–1.0)	0.021	0.8 (0.7–0.9)	0.006
Never a BMC	25825	1263 (4.9)				
Ever a BMC in Central Appalachia	2401	154 (6.4)	4.5 (3.0–6.6)	<0.001	3.4 (2.3–5.0)	<0.001
Ever a BMC in US excluding Central Appalachia	2093	30 (1.4)				

95% CI, 95% confidence interval; CWHSP, Coal Workers’ Health Surveillance Program; PR, prevalence ratio. ^a^PR adjusted for underground mining tenure. ^b^Central Appalachia includes Kentucky, Virginia, and West Virginia.

**Table 3:** Prevalence of progressive massive fibrosis (PMF) and associated prevalence ratios by region and occupational history among U. S. underground coal miners, CWHSP, 1991–2024.

	Progressive massive fibrosis (PMF)					
	*N*	*n*	Unadjusted PR (95% CI)	*p*-value	PR (adjusted)^a^	*p*-value
US underground coal miners						
Central Appalachia^b^	14284	182 (1.3)	15.7 (9.0–27.6)	<0.001	10.0 (5.7–17.5)	<0.001
US excluding Central Appalachia	16035	13 (0.1)				
Roof bolter status						
Ever roof bolters	13072	103 (0.8)	1.5 (1.1–2.0)	0.0064	1.4 (1.1–1.9)	0.017
Never roof bolters	17247	92 (0.5)				
Ever a roof bolter in Central Appalachia	7053	98 (1.4)	16.7 (6.8–41.1)	<0.001	12.0 (4.9–29.4)	<0.001
Ever a roof bolter in US excluding Central Appalachia	6019	5 (0.1)				
Continuous miner operator (CMO) status						
Ever a CMO	7871	117 (1.5)	4.3 (3.2–5.7)	<0.001	3.0 (2.3–4.0)	<0.001
Never a CMO	22448	78 (0.4)				
Ever a CMO in Central Appalachia	4011	107 (2.7)	10.3 (5.4–19.7)	<0.001	8.0 (4.2–15.3)	<0.001
Ever a CMO in US excluding Central Appalachia	3860	10 (0.3)				
Shuttle car operator status						
Ever a shuttle car operator	8641	63 (0.7)	1.2 (0.9–1.6)	0.238	0.9 (0.7–1.2)	0.581
Never a shuttle car operator	21678	132 (0.6)				
Ever a shuttle car operator in Central Appalachia	4819	60 (1.3)	15.9 (5.0–50.5)	<0.001	11.7 (3.7–37.0)	<0.001
Ever a shuttle car operator in US excluding Central Appalachia	3822	3 (0.1)				
Laborer status						
Ever a laborer	9430	43 (0.5)	0.6 (0.5–0.9)	0.007	0.6 (0.4–0.8)	0.001
Never a laborer	20889	152 (0.7)				
Ever a laborer in Central Appalachia	4023	41 (1.0)	27.6 (6.7–113.8)	<0.001	19.1 (4.7–78.4)	<0.001
Ever a laborer in US excluding Central Appalachia	5407	2 (0.04)				
Belt man/Conveyor man/Belt cleaner (BMC) status						
Ever a BMC	4494	16 (0.4)	0.5 (0.3–0.9)	0.012	0.5 (0.3–0.8)	0.010
Never a BMC	25825	179 (0.7)				
Ever a BMC in Central Appalachia	2401	15 (0.6)	13.1 (1.7–98.9)	0.013	9.9 (1.3–74.4)	0.026
Ever a BMC in US excluding Central Appalachia	2093	1 (0.1)				

95% CI, 95% confidence interval; CWHSP, Coal Workers’ Health Surveillance Program; PR, prevalence ratio. ^a^PR adjusted for underground mining tenure. ^b^Central Appalachia includes Kentucky, Virginia, and West Virginia.

### Prevalence by mining occupations

Occupational differences were observed for CWP and PMF. For CWP, roof bolters and CMOs had higher prevalence compared with miners who never held these roles [adjusted PR = 1.4 (95% CI: 1.3–1.6) and 1.8 (95% CI: 1.6–2.0), respectively]. In contrast, working as a shuttle car operator had lower CWP prevalence after adjustment (PR = 0.9; 95% CI: 0.8–1.0), as well as laborers and belt man/conveyor workers with lower prevalence (PR = 0.7; 95% CI: 0.6–0.8 and PR = 0.8; 95% CI: 0.7–0.9, respectively). For PMF, CMOs and roof bolters also had higher prevalence [adjusted PR = 3.0 (95% CI: 2.3–4.0) and 1.4 (95% CI: 1.1–1.9), respectively], whereas no association was observed for shuttle car operators (PR = 0.9; 95% CI: 0.7–1.2), and lower prevalence was observed among laborers (PR = 0.6; 95% CI: 0.4–0.8) and belt man/conveyor workers (PR = 0.5; 95% CI: 0.3–0.8).

When stratified by region, significantly elevated prevalence of both CWP and PMF was observed across all occupations in Central Appalachia compared with other U. S. regions. For CWP, adjusted prevalence ratios ranged from 3.3 [95% CI: 2.6–4.2] among laborers to 5.1 [95% CI: 4.1–6.3] among CMOs. For PMF, the magnitude of association was notably greater, with adjusted prevalence ratios ranging from 8.0 [95% CI: 4.2–15.3] among CMOs to 19.1 [95% CI: 4.7–78.4] among laborers.

## Discussion

In this analysis of more than 30,000 underground coal miners participating in the CWHSP during 1991–2024, we observed significant differences in the prevalence of CWP and PMF by underground mining occupation and geographic region. CMOs and roof bolters experienced the highest prevalence of both CWP and PMF after adjustment for underground mining tenure, while miners in Central Appalachia had markedly higher disease prevalence across all occupational groups. Together, these findings reinforce longstanding concerns regarding elevated respirable coal mine dust and respirable crystalline silica exposures among miners performing cutting, drilling, and extraction activities in underground coal mines (20).

The observed occupational patterns are consistent with prior exposure-based investigations demonstrating that CMOs and roof bolters routinely experience some of the highest respirable dust concentrations in underground coal mining environments (10). Continuous miner operations involve direct coal cutting and extraction, including cutting through rock strata that may contain high concentrations of crystalline silica. Roof bolting similarly requires drilling through overhead rock, generating silica-containing dust. Previous dust sampling studies have documented elevated exposures among these occupations, particularly in thin-seam mining conditions common in Central Appalachia (21). The strong association between CMO status and PMF in this study further supports growing evidence that crystalline silica exposure plays a major role in the development of severe and rapidly progressive pneumoconiosis (4).

Although roof bolters and CMOs demonstrated the highest prevalence ratios, elevated disease prevalence was observed across multiple underground occupations, particularly in Central Appalachia. Even occupations with lower adjusted prevalence ratios, such as laborers and belt man/conveyor workers, experienced substantially greater prevalence of both CWP and PMF in Central Appalachia compared with miners in other U. S. regions. These findings suggest that hazardous dust exposures remain pervasive throughout the underground mining environment and are not confined solely to workers directly engaged in cutting and drilling tasks.

The regional disparity observed in this analysis is striking and consistent with previous reports documenting the resurgence of severe pneumoconiosis in Central Appalachia (9, 12, 22). After adjustment for underground mining tenure, miners in Central Appalachia had nearly four times the prevalence of CWP and 10 times the prevalence of PMF compared with miners working elsewhere in the United States. Several factors likely contribute to this disparity, including mining of thinner coal seams requiring increased cutting through crystalline silica-rich rock strata, high-production underground mining methods, and cumulative exposure histories accrued over decades. Prior radiographic and pathology investigations have suggested and demonstrated substantial crystalline silica and silicate particle burden in the lungs of miners with rapidly progressive pneumoconiosis, implicating respirable crystalline silica exposure as an important driver of contemporary severe disease (4, 22, 23).

Increasing underground mining tenure was strongly associated with greater radiographic severity of pneumoconiosis. Most PMF cases occurred among miners with more than 25 years of underground mining tenure, consistent with the cumulative nature of dust-related lung disease. However, the occurrence of PMF among miners with fewer than 20 years of underground mining is concerning and aligns with previous reports of rapidly progressive disease among relatively younger miners in Central Appalachia. These findings suggest that some contemporary mining environments may involve sufficiently intense dust exposures that accelerate disease progression (24).

This study extends previous work examining respirable dust concentrations in underground coal mines by linking occupational histories collected through a national surveillance program with radiographic evidence of disease. The large sample size, inclusion of miners from multiple U. S. mining regions, and standardized radiographic classification methods strengthen the findings. However, several limitations should be considered. Participation in the CWHSP is voluntary, and participating miners may not fully represent all U. S. underground coal miners. Since this analysis was restricted to working miners, the findings may be affected by the healthy worker survivor effect. Occupational histories were self-reported and miners frequently worked in multiple occupations during their careers, limiting attribution of disease risk to a single job category. Occupational classification was based on whether miners had ever worked in specific occupations and did not account for each occupation-specific tenure or the intensity of respirable dust exposure. Thus, miners within the same occupational category may have had different cumulative exposures. Prevalence ratios were adjusted for total mining tenure, and geographic differences were examined separately given the higher prevalence of CWP documented among miners in Central Appalachia. However, residual confounding by other factors, including age and sex, may remain. In addition, quantitative individual-level dust exposure data were not available. Therefore, the observed occupational associations should not be interpreted as evidence of an exposure-response relationship. There were no missing data for the variables included in the analysis.

Despite these limitations, the findings have important implications for prevention and surveillance. The persistence of elevated pneumoconiosis prevalence among occupations historically recognized as highly dust-exposed demonstrates the continued need for rigorous dust control, exposure monitoring, and medical surveillance. The 2024 MSHA Silica Rule would lower the permissible exposure limit for respirable crystalline silica in coal mines (25). In accordance with a federal court order, MSHA has delayed implementation of the Rule and will enforce existing standards (26). Continued engineering controls, enhanced compliance monitoring, and expanded participation in surveillance programs remain critical to preventing future cases of severe pneumoconiosis.

In conclusion, from 1991 to 2024, working as a continuous miner operator and roof bolter was associated with elevated prevalence of CWP and PMF, particularly among underground coal miners in Central Appalachia. These findings highlight the enduring burden of occupational lung disease among underground coal miners and underscore the importance of continued efforts to reduce respirable coal mine dust in high-risk mining occupations.

## Data Availability

The datasets presented in this article are not readily available because Aggregated data is available by request. Requests should be directed to Nirmala Myers nmyers@cdc.gov.
