# Supplementary material for: Assessment of pneumoconiosis by dusty job type among underground coal miners participating in the Coal Workers’ Health Surveillance Program,1991–2024
Source: Front Public Health. 2026 Oct 20;14:1970741. doi: 10.3389/fpubh.2026.1970741 (PMC13727509; doi:10.3389/fpubh.2026.1970741)
Supplement: Supplementary file 1 [file Table_1.pdf]

Assessment of pneumoconiosis by dusty job type among underground coal miners participating in the Coal Workers' Health Surveillance Program, 1991–2024.

Nirmala T. Myers, PhD, David J. Blackley, DrPH, Laura Reynolds, MPH, BSN, RN, A. Scott Laney, PhD, MPH, and Noemi B. Hall, PhD.
Surveillance Branch, Respiratory Health Division, National Institute for Occupational Safety and Health, Centers for Disease Control and Prevention, Morgantown, WV, United States

SI. Table 1. Underground coal occupation groups using Mine Safety and Health Administration (MSHA) Dust Samples Job Codes

Underground Coal Occupation Groups	MSHA Job Codes [a]
Auger	010, 017, 018, 055, 070, 071
Belt man / Conveyor Man / Belt Cleaner	001, 101, 154, 201
Clerk (Timekeeping)	497
Coal Driller Operator and Helper	033, 034
Coal Truck Driver	376
Continuous Miner Operator and Helper	035, 036
Cutting Machine Operator and Helper	037, 038
Driller / Blaster / Shotfirer	007, 031, 307, 333, 334, 356, 383, 384
Electrician and Helper	002, 003, 102, 103
Engineer	456
Fan Attendant	015, 115
Heavy Equipment Operator	368
Laborer	016, 116, 316
Longwall	040, 041, 044, 051, 052, 060, 061, 064
Mechanic and Helper	004, 005, 104, 105
Motorman	074, 269
Rock Duster	006, 106
Roof Bolter	012, 014, 019, 046, 047, 048, 146

Underground Coal Occupation Groups	MSHA Job Codes [a]
Shuttle Car Operator	050, 073, 250
Stopping Builder / Brattice Man	008, 032, 108
Supply Man	009, 109
Warehouse	498

[a] MSHA codes are available at https://arlweb.msha.gov/drs/CODES/0XX.HTM
